# Effect of dietary energy levels and phase feeding by protein levels on growth performance, blood profiles and carcass characteristics in growing-finishing pigs

**DOI:** 10.1186/s40781-016-0119-z

**Published:** 2016-10-24

**Authors:** J. S. Hong, G.I. Lee, X. H. Jin, Y. Y. Kim

**Affiliations:** 1 School of Agricultural Biotechnology, Seoul National University, Seoul, South Korea; 2 College of Agriculture and Life Sciences, Seoul National University, Seoul, 151-921 South Korea

**Keywords:** Energy, Growing-finishing pig, Growth performance, Phase feeding, Protein

## Abstract

**Background:**

Providing of insufficient nutrients limits the potential growth of pig, while feeding of excessive nutrients increases the economic loss and causes environment pollution. For these reasons, phase feeding had been introduced in swine farm for improving animal production. This experiment was conducted to evaluate the effects of dietary energy levels and phase feeding by protein levels on growth performance, blood profiles and carcass characteristics in growing-finishing pigs.

**Methods:**

A total of 128 growing pigs ([Yorkshire × Landrace] × Duroc), averaging 26.62 ± 3.07 kg body weight, were assigned in a 2 × 4 factorial arrangement with 4 pigs per pen. The first factor was two dietary energy level (3,265 kcal of ME/kg or 3,365 kcal of ME/kg), and the second factor was four different levels of dietary protein by phase feeding (1growing(G)-2finishing(F) phases, 2G-2F phases, 2G-3F phases and 2G-3F phases with low CP requirement).

**Results:**

In feeding trial, there was no significant difference in growth performance. The BUN concentration was decreased as dietary protein level decreased in 6 week and blood creatinine was increased in 13 week when pigs were fed diets with different dietary energy level. The digestibility of crude fat was improved as dietary energy levels increased and excretion of urinary nitrogen was reduced when low protein diet was provided. Chemical compositions of longissimus muscle were not affected by dietary treatments. In backfat thickness (P_2_) at 13 week, pigs fed high energy diet had thicker backfat thickness (*P* = 0.06) and pigs fed low protein diet showed the trend of backfat thinness reduction (*P* = 0.09). In addition, water holding capacity was decreased (*P* = 0.01) and cooking loss was increased (*P* = 0.07) as dietary protein level reduced. When pigs were fed high energy diet with low subdivision of phase feeding, days to 120 kg market weight was reached earlier compared to other treatments.

**Conclusion:**

Feeding the low energy diet and subdivision of growing-finishing phase by dietary protein levels had no significant effect on growth performance and carcass characteristics. Also, phase feeding with low energy and low protein diet had no negative effects on growth performance, carcass characteristics but economical profits was improved.

## Background

In livestock industry, providing adequate nutrients has been noted as the most important factor to achieve efficient and profitable animal production. Providing of insufficient nutrients limits the potential growth and production of animal, while feeding of excessive nutrients reduces the economic profitability and causes environment pollution [18, 19, 31]. Providing adequate nutrients to animal is difficult because nutrient requirements of animal continuously are changed with aging [29]. Thus the concept of phase feeding has been introduced in swine production, which divides the growth period into several phases and provide feed designated to each phase.

Subdivision of feeding phases needs careful considerations because feeding one diet causes oversupply of nutrients in some period and results in deficiency of nutrients in other period [7, 14, 22]. However, importance of nutrient requirement for growing · finishing pigs was underestimated and overlooked compared to those of younger pigs although the growing-finishing period account for approximately 60 % of pig’s lifetime [25, 29] with about 80 % of total feed consumption. In growing period, pig shows the highest weight gain in lifetime for extensive muscle development [41]. Changing diet with adequate dietary protein and amino acid contents can be the most important factor to maximizing the muscle development because protein and amino acid requirements are changing drastically in this period [33]. However, fat deposition of pig is much greater than protein deposition in finishing period [41], which resulted in carcass fatness and poor pork quality. Although these differences are made from continuous changes of nutrient requirements, NRC [30] recommended 2 growing and 2 finishing periods, respectively compared to 1 growing and 2 finishing periods in 1998 version of NRC. Moreover, NRC of 1998 and 2012 versions established four or three feeding phases for younger pigs until reaching 20 kg and 25 kg body weight, respectively. Therefore, more subdivision of growing-finishing period through phase feeding takes potential benefits in nutrient efficiency and maximizing growth performance, inducing high profit for wine producers.

Therefore, present study was conducted to evaluate the subdivision of growing-finishing phase based on dietary protein levels in different dietary energy levels on growth performance, blood profiles and carcass characteristics.

## Methods

### Animal and management

All experimental procedures involving animals were conducted in accordance with the Animal Experimental Guidelines provided by the Seoul National University Institutional Animal Use and Care Committee (SNUIAUCC; SNU-160513-1).

A total of 128 crossbred pigs ([Yorkshire × Landrace] × Duroc) with an average body weight of 26.62 ± 3.07 kg were used for 13 weeks feeding trial. Pigs were reared in experimental farm of Seoul National University located in Suwon, Gyeonggi-Do. Two male and two female pigs were assigned to each pen of growing facility based on body weight. Pigs were reared in growing (1.30 × 2.50 m^2^) facilities for 6 weeks and then moved to finishing (1.60 × 3.10 m^2^) facilities for the rest 7 weeks. Feed and water were provided *ad libitum* during the whole experimental period by a 4 hole stainless feeder and a nipple installed in each pen.

### Experimental design and diet

Experimental pigs were allotted to 2 × 4 factorial arrangement in randomized complete block (RCB) design with 3 replicates and 4 pigs per pen. The first factor was two levels of dietary energy density (3,265 kcal of ME/kg or 3,365 kcal of ME/kg), and the second factor was four dietary protein levels based on subdivision of growing-finishing phases (CON: 1 phase and 2 phases for growing and finishing pigs; P4: 2 phases and 2 phases for growing and finishing pigs; P5: 2 phases and 3 phases for growing and finishing pigs; LP5: 2 phases and 3 phases for growing and finishing pigs with lower dietary protein levels). The standard of dividing growing-finishing phase is determined to regression equation of nutrient requirement from NRC [29]. The CP requirements and body weight ranges presented in NRC [29] were used to make the regression equation and line. As a result of regression equation, CP requirement for each phase was determined. Growing phase1: CP 18.0 %/BW (25-40 kg), growing phase2: CP 16.3 %/BW (40-60 kg), finishing phase1: CP 14.9 %/BW (60-80 kg), finishing phase2: CP 13.9 %/BW (80-100 kg), finishing phase3: CP 13.2 %/BW (100-120 kg). The CP requirement of LP5 treatment was determined from value of final body weight in each phase (40 kg, 60 kg, 80 kg, 100 kg, 120 kg).

$$ \mathrm{Y} = -4.001 \ln \left(\times \right)+31.947\ \left({\mathrm{R}}^2 = 0.9912\right) $$

Experimental diets were formulated for 5 phases, including growing phase1 (0-3 week), growing phase2 (4-6 week), finishing phase1 (7-9 week), finishing phase2 (10-12 week), finishing phase3 (13 week). All nutrients of experimental diets except CP and lysine were met or exceeded the nutrient requirement of NRC [29]. Chemical composition of experimental diets were presented in Tables 1, 2 and 3.

**Table 1:** Experimental design of subdivision of growing-finishing period by protein levels

Treatment	0-3 week	3-6 week	6-9 week	9-12 week	12-13 week
CON (CP level)	Growing 1 (18.0 %)		Finishing 1 (15.5 %)	Finishing 2 (13.2 %)	
P4 (CP level)	Growing 1 (18.0 %)	Growing 2 (16.3 %)	Finishing 1 (15.5 %)	Finishing 2 (13.2 %)	
P5 (CP level)	Growing 1 (18.0 %)	Growing 2 (16.3 %)	Finishing 1 (14.9 %)	Finishing 2 (13.9 %)	Finishing 3 (13.2 %)
LP5 (CP level)	Growing 1 (17.2 %)	Growing 2 (15.6 %)	Finishing 1 (14.4 %)	Finishing 2 (13.5 %)	Finishing 3 (12.8 %)

**Table 2:** Chemical compositions of experimental diets (growing phase)

	Growing 1 phase (0-3 week)		Growing 2 phase (4-6 week)		
Treatment	CON, P4, P5	LP5	CON	P4, P5	LP5
ME (Kcal/kg)^b^	ME 3,265 or 3,365 kcal/kg				
Crude protein (%)^b^	18.0	17.2	18.0	16.3	15.6
Crude protein (%)^a^	17.7	17.0	18.3	16.5	15.5
Crude fat (%)^a,c^	2.6/4.2	2.6/4.4	3.2/4.2	3.0/4.0	2.7/4.5
Lysine (%)^b^	0.95	0.88	0.95	0.82	0.77
Methionine (%)^b^	0.26	0.25	0.26	0.25	0.24
Tryptophan (%)^b^	0.22	0.21	0.22	0.20	0.19
Tryptophan (%)^b^	0.72	0.69	0.72	0.65	0.63
Calcium (%)^b^	0.60	0.57	0.60	0.54	0.52
Total phosphorus (%)^b^	0.50	0.50	0.50	0.47	0.45

^a^analyzed value, ^b^calculated value, ^c^Analyzed crude fat content: ME 3,265 kcal diet/ME 3,365 kcal diet

**Table 3:** Chemical compositions of experimental diets (finishing phase)

	Finishing 1 phase			Finishing 2 phase		Finishing 3 phase		
Treatment	CON, P4	P5	LP5	CON, P4	P5	LP5	CON, P4, P5	LP5
ME (Kcal/kg)^b^	ME 3,265 or 3,365 kcal/kg							
Crude protein (%)^b^	15.5	14.9	14.4	13.2	13.9	13.5	13.2	12.8
Crude protein (%)^a^	15.7	15.0	14.5	12.9	14.0	13.6	13.5	12.6
Crude fat (%)^a,c^	2.1/3.8	1.8/3.8	2.3/3.4	2.8/5.3	2.7/5.0	2.9/5.3	3.8/5.6	3.3/5.6
Lysine (%)^b^	0.75	0.72	0.68	0.60	0.65	0.62	0.60	0.57
Methionine (%)^b^	0.24	0.23	0.23	0.22	0.23	0.22	0.22	0.22
Tryptophan (%)^b^	0.18	0.18	0.17	0.15	0.16	0.15	0.15	0.14
Tryptophan (%)^b^	0.62	0.60	0.58	0.53	0.55	0.54	0.53	0.51
Calcium (%)^b^	0.50	0.50	0.48	0.45	0.46	0.45	0.45	0.43
Total phosphorus (%)^b^	0.45	0.44	0.43	0.40	0.41	0.41	0.40	0.38

^a^analyzed value, ^b^calculated value, ^c^Analyzed crude fat content: ME 3,265 kcal diet/ME 3,365 kcal diet

### Growth performance

Body weight and feed consumption were recorded at 0, 3, 6, 9, 12 and 13 weeks to calculate average daily gain (ADG), average daily feed intake (ADFI) and gain to feed ratio (G/F ratio).

### Blood profiles

Blood samples were taken from the jugular vein of randomly selected six pigs in each treatment for measuring blood urea nitrogen (BUN) and blood creatinine when after pigs were fasten 3 h. Collected blood samples were centrifuged for 15 min at 3,000 rpm on 4 °C (Eppendorf centrifuge 5810R, Germany). The sera were carefully transferred to 1.5 ml plastic tubes and stored at –20 °C until later analysis. Total BUN concentration was analyzed using a blood analyzer (Ciba-Corning model, Express Plus, Ciba Corning Diagnostics Co.). Creatinine concentration was measured by kinetic colorimetry assay using a blood analyzer (Modular analytics, PE, Roche, Germany)

### Digestibility trial

A total of 24 crossbred growing barrows, averaging 49.37 ± 2.97 kg body weight, were allotted to individual metabolic crate in completely randomized design (CRD) with 4 replicates to evaluate the nutrient digestibility and nitrogen retention. Experimental diets of growing phase 2 (CON: 18 % crude protein, P4 = P5: 16.3 % crude protein, LP5: 15.6 % crude protein) were provided to each treatment animals. Total collection method was used for the apparent nutrient digestibility. After a 5 days adaptation period, 5 days of collection period was followed. To determine the first and last day of collection days, 2 g of ferric oxide and chromium oxide were added in the first and last experimental diet as selection marker, respectively. During the experimental period, all pigs were fed the experimental diets twice a day at 7:00 and 19:00, and water was provided *ad libitum*. Total urine was collected daily in a plastic container containing 50 ml of 4 N H_2_SO_4_ to avoid nitrogen evaporation and frozen during the 5 days of collection period for nitrogen retention analysis. Collected feces and urine samples were stored –20 °C until analysis. Collected excreta were pooled and dried in an air-forced drying oven at 60 °C for 72 h, and then ground into 1 mm particles in a Wiley mill for chemical analysis include moisture, protein, fat and ash contents [1].

### Carcass characteristics

At the end of experiment, 3 pigs from each treatment group were selected and slaughtered for the carcass analysis. Pork samples were collected from nearby 10th rib on right side of carcass. Because of chilling procedure, 30 min after slaughter was regarded as initial time. The pH was measured at 0, 3, 6, 12 and 24 h and meat color of longissimus muscle was determined at initial time and 24 h after initial time. The pH was measured using a pH meter (Model 720, Thermo Orion, U.S.A.) and pork color was determined by CIE color L*, a*, and b* values using a CR300 (Minolta Camera Co., Japan). Chemical analysis of pork samples were conducted by the method of AOAC [1]. Water holding capacity of pork was measured by centrifuge method. Three samples of longissimus muscles were ground and sampled in filter tube, than heated in water bath at 80 °C for 20 min and centrifuged for 10 min at 2,000 rpm at 10 °C (Eppendorf centrifuge 5810R, Germany). To calculate the cooking loss, longissimus muscles were packed with polyethylene bag and heated in water bath until core temperature reached 72 °C and weighed before and after cooking. After heated, samples are cored (0.5 in. in diameter) parallel to muscle fiber and the cores were used to measure the shear force using a salter (Warner Barzler Shear, USA). Water holding capacity, shear force and cooking loss of pork were analyzed in Foundation of Agri. Tech. Commercialization & Transfer. The amino acid contents in loin meat were determined by ion-exchange chromatography (Amino Acid Analyzer L-8900, Hitachi, Tokyo, Japan) with post-column derivatization with ninhydrin. Performic acid was used in oxidizing amino acids and neutralized with sodium citrate dihydrate, and then hydrolyzed with 6 N HCl for 22 h at 110 °C to be liberated from the protein. Amino acids were quantified with the internal standard method (amino acid mixture standard solution Type H, Wako Chemical, Osaka, Japan; L-cysteic acid, Tockyo Chemical Industry, Tokyo, Japan; DL-methionine sulfone, Sigma, Missouri, US) by measuring the absorption of reaction products with ninhydrin at 570 nm.

Backfat thickness at P_2_ position (mean value from both sides of the last rib and 65 mm away from the backbone) of finishing pigs was measured using ultrasound device (Lean-meter, Renco Corp., Minneapolis, US) at the end of feeding trial (13 week).

### Economical analysis

As the experimental pigs were reared in the same environmental condition, economical efficiency was calculated using only the feed cost without considering other factors. The total feed cost and feed cost (won) per body weight gain (kg) was calculated using amount of the total feed intake and feed price. The days to reaching market weight (120 kg) was estimated from the body weight at the end of feeding trial and ADG of 12-13 week. A total of 48 finishing pigs were slaughtered at slaughter house and all carcass was graded by livestock product grading standard (Korea Institute for Animal Products Quality Evaluation).

### Statistical analysis

All data were analyzed using PROC MIXED procedures of SAS. Each pen was regarded as an experimental unit in feeding trial, and individual pig was used as an experimental unit in blood profiles, digestibility trial and carcass analysis. The statistical model included two main effects, dietary energy level and subdivision of phase by dietary protein level. PDIFF option of SAS was used to evaluate the effect of main factors and their interaction. Significance for treatment effects was reported at *P* ≤ 0.05, with a trend between *P* ≥ 0.05 and *P* ≤ 0.10.

## Results and discussion

### Growth performance

The effect of dietary energy levels and phase feeding by protein levels on growth performance was presented in Table 4. During the whole experimental period, dietary energy density and phase feeding by protein level did not show any significant differences in body weight, average daily gain (ADG), average daily feed intake (ADFI) and gain/feed ratio (G/F ratio). Numerous researches presented the benefits of increasing energy density in feed with improvement of weight gain and feed efficiency by lower feed intake [10, 32, 35]. However, in current study, dietary energy level had little effects on the growth performance of growing ·finishing pigs. Many researchers also found that increasing energy density had no influences on growth performance in growing ·finishing pigs when feed contained sufficient energy for potential growth [9, 17, 27]. Therefore, 3,265 kcal of ME/kg in this study was sufficient energy for growth of growing ·finishing pigs.

**Table 4:** Effects of dietary energy and protein levels on growth performance in growing-finishing pigs^a^

Criteria	Treatments						SEM^b^	*P*-value		
	ME 3,265	ME 3,365	CON	P4	P5	LP5		E	P	E×P
Body weight, kg										
Initial	26.27	26.35	26.25	26.31	26.32	26.37		-	-	-
3 weeks	43.62	43.41	43.14	42.64	44.39	43.89	0.740	0.90	0.88	0.86
6 weeks	61.39	61.11	61.45	59.90	62.33	61.32	0.893	0.89	0.85	0.85
9 weeks	80.98	79.43	81.20	78.42	80.40	80.80	0.998	0.46	0.79	0.44
12 weeks	100.67	99.86	101.27	98.06	100.60	101.12	1.134	0.74	0.77	0.66
13 weeks	107.75	107.64	107.67	105.80	108.89	108.43	1.190	0.97	0.84	0.58
ADG, g										
0-3 weeks	826	812	804	778	861	835	16.1	0.66	0.30	0.28
3-6 weeks	846	843	872	823	855	830	17.8	0.94	0.79	0.84
6-9 weeks	933	872	941	882	860	928	22.0	0.77	0.56	0.61
9-12 weeks	938	973	956	936	962	968	20.0	0.17	0.52	0.28
12-13 weeks	1,012	1,112	913	1,106	1,185	1,044	50.4	0.42	0.96	0.60
0-6 weeks	836	828	838	800	858	832	13.6	0.33	0.30	0.55
6-13 weeks	946	950	943	937	950	962	11.2	0.89	0.90	0.41
0-13 weeks	895	894	895	874	908	902	10.6	0.92	0.72	0.41
ADFI, g										
0-3 weeks	1,644	1,678	1,672	1,606	1,751	1,616	42.8	0.71	0.67	0.70
3-6 weeks	2,177	2,138	2,202	2,081	2,246	2,102	48.4	0.71	0.64	0.94
6-9 weeks	2,583	2,595	2,702	2,514	2,599	2,541	48.3	0.98	0.61	0.90
9-12 weeks	2,903	2,875	2,924	2,931	2,957	2,743	42.1	0.92	0.61	0.96
12-13 weeks	3,035	3,252	3,146	3,274	3,069	3,085	53.0	0.73	0.25	0.19
0-6 weeks	1,911	1,908	1,937	1,843	1,999	1,859	42.3	0.49	0.22	0.43
6-13 weeks	2,785	2,808	2,860	2,801	2,819	2,705	34.5	0.97	0.41	0.89
0-13 weeks	2,381	2,393	2,434	2,359	2,440	2,314	35.1	0.99	0.50	0.92
G/F ratio										
0-3 weeks	0.510	0.487	0.483	0.485	0.497	0.530	0.0103	0.28	0.38	0.55
3-6 weeks	0.391	0.397	0.399	0.395	0.382	0.400	0.0073	0.69	0.84	0.60
6-9 weeks	0.363	0.340	0.350	0.355	0.333	0.369	0.0099	0.76	0.71	0.67
9-12 weeks	0.326	0.339	0.327	0.322	0.329	0.354	0.0082	0.26	0.66	0.41
12-13 weeks	0.337	0.342	0.292	0.337	0.392	0.339	0.0174	0.45	0.57	0.67
0-6 weeks	0.441	0.437	0.436	0.435	0.432	0.453	0.0066	0.35	0.46	0.77
6-13 weeks	0.342	0.339	0.331	0.335	0.338	0.357	0.0052	0.98	0.31	0.77
0-13 weeks	0.378	0.375	0.369	0.371	0.373	0.392	0.0050	0.92	0.33	0.78

^a^A total 128 crossbred pigs was fed from average initial body 26.62 ± 3.07 kg and the average final body weight was 107.7 kg. ^b^Standard error of mean

In phase feeding by protein levels, increasing subdivision of growing-finishing phase did not influence on growth performance. Similar to this observation, some researchers indicated that the number of phases did not have any effect on growth performance of growing ·finishing pigs [8, 24, 26]. Since consumption of protein above requirement led to excreta [7], pig utilized the constant level of crude protein [8]. In addition, LP5 group which fed diet contained lower protein also showed similar growth performance with P5 group. Wahlstrom and Libal [40] and Tjong et al. [38] demonstrated that supplementation of diet with lower crude protein than NRC requirement had no detrimental effects on growth performance. In the study of Carpenter et al. [5], limitation of protein level in diet had no negative effects on growth performance during finishing period. Consequently, the results of current study demonstrated that pigs fed diet contained 3,265 kcal ME/kg of energy density and reduced crude protein level had no detrimental effects on growth performance compared with higher energy density or crude protein contents.

### Blood profiles

The effect of dietary energy levels and phase feeding by protein levels on blood urea nitrogen (BUN) and blood creatinine concentration were shown in Table 5. At 6 week, reduction of BUN was observed by subdivision of phases especially when pigs were fed low protein diet (*P* < 0.01). Similarly, Lee et al. [26] represented that two or three phase feedings resulted less BUN concentration compared to that of one phase feeding. Excessive consumption of protein decreased the availability of protein [18] and increased the excretion of the nitrogen as urea form [15]. Thus, increase of BUN concentration indicated that excessive amino acids are inefficiently metabolized and circulated in the blood before excretion [18]. The result of current study was in agreement with previous researches which observed reduction of BUN concentration with supplementation of lower crude protein diet [6, 7, 13, 18]. In the results of growth performance, increasing subdivision of phase feeding or reduced dietary crude protein level had no detrimental effects. Therefore, decrease of BUN concentration at 6 week indicated that efficiency of crude protein utilization can be improved by phase feeding or reduced dietary crude protein level especially in growing period.

**Table 5:** Effects of dietary energy and protein levels on blood profiles in growing · finishing pigs^1^

Criteria	Treatments						SEM^2^	*P*-value		
	ME 3,265	ME 3,365	CON	P4	P5	LP5		E	P	E × P
BUN, mg/dL										
Initial	10.28	10.28	10.28	10.28	10.28	10.28	-	-	-	-
3 weeks	11.61	11.14	11.95	11.76	11.60	10.18	0.285	0.41	0.12	0.78
6 weeks	10.65	9.90	12.30^A^	10.10^B^	10.10^B^	8.60^B^	0.334	0.21	0.01	0.30
9 weeks	9.45	10.14	10.14	10.46	9.45	9.13	0.258	0.18	0.25	0.64
12 weeks	11.42	11.69	10.85	12.70	10.80	11.87	0.329	0.69	0.15	0.83
13 weeks	11.68	10.65	10.17	10.81	12.23	11.45	0.378	0.18	0.27	0.85
Creatinine, mg/dL										
Initial	1.00	1.00	1.00	1.00	1.00	1.00	-	-	-	-
3 weeks	0.82	0.82	0.72	0.78	0.92	0.84	0.030	1.00	0.12	0.34
6 weeks	0.96	0.89	0.89	0.90	0.94	0.98	0.025	0.17	0.58	0.80
9 weeks	1.18	1.20	1.11	1.22	1.19	1.25	0.032	0.71	0.52	0.54
12 weeks	1.44	1.40	1.38	1.40	1.38	1.53	0.030	0.58	0.26	0.30
13 weeks	1.45^a^	1.32^b^	1.31	1.45	1.32	1.47	0.033	0.05	0.17	0.98

^1^Least squares means of 6 observations per treatment

^2^Standard error of mean

^AB^means with different superscripts in the same row significantly differ (*P* < 0.05)

^ab^means with different superscripts in the same row significantly differ (0.05 ≤ *P* < 0.10)

Blood creatinine has positive correlation with content of total muscle [3] and striated muscle [34]. At 13 week, when pigs were fed low energy (3,265 kcal of ME/kg) diet had higher blood creatinine concentration than pigs fed high energy (3,365 kcal of ME/kg) diet (*P* = 0.05). Low energy diet resulted in increased percent yield of lean cuts [39] and high energy diet increased carcass fatness [28, 32]. This observation demonstrated that increasing energy density in diet stimulated the rate of lipid accumulation and reduced the lean percentage in rats [36]. Therefore, pigs fed low energy diet (3,265 kcal of ME/kg) had greater blood creatinine concentration than pigs fed high energy diet because of more lean and less fat depositions.

### Nutrient digestibility

The effect of dietary energy levels and phase feeding by protein levels on nutrient digestibility and nitrogen retention was presented in Table 6. Significant improvement of crude fat digestibility was observed when pigs were fed greater energy treatment group (*P* = 0.02). However, there was no significant difference in digestibilities of dry matter, crude protein and crude ash. Improvement of fat digestibility in current study might be resulted from the highly digestible soy oil which was included in feed formulation to increase energy density of high energy density diet (3,365 kcal of ME/kg). Just [20] represented that increase of fatty acids digestibility by inclusion of plant-derived oils and Kim et al. [23] demonstrated that fat digestibility of extracted corn oil was higher than that of intact sources of oil from high-oil corn, DDGS, corn germ, or HP DDG.

**Table 6:** Effects of dietary energy and protein levels on nutrient digestibility and nitrogen retention in growing · finishing pigs^1^

Criteria	Treatments^3^					SEM^2^	*P*-value		
	ME 3,265	ME 3,365	CON	P4(P5)	LP5		E	P	E×P
Nutrient digestibility, %									
Dry matter	90.33	90.44	90.63	90.21	90.31	0.233	0.84	0.79	0.78
Crude protein	87.60	87.53	88.70	87.20	86.80	0.409	0.99	0.19	0.46
Crude ash	60.52	59.35	59.55	58.19	62.06	1.078	0.61	0.38	0.64
Crude fat	71.00^B^	76.80^A^	77.45	71.94	72.33	1.358	0.02	0.12	0.28
N-retention, g/d									
N-intake	116.99	117.37	127.04	114.70	109.82	1.832			
N-feces	14.43	14.53	14.35	14.69	14.40	0.460	0.93	0.96	0.48
N-urine	71.25	70.69	81.55^A^	71.69^A^	59.68^B^	2.628	0.89	0.01	0.58
N-retention^4^	31.31	32.16	31.14	28.32	35.75	2.175	0.82	0.26	0.65
N-retention^5^, %	27.02	27.49	24.51^b^	24.68^ab^	32.57^a^	1.747	0.88	0.08	0.67

^1^A total of 24 barrow and initial body weight 49.37 ± 2.97 kg

^2^CON: CP 18.0 %, P4(P5): CP 16.3 %, LP5: CP 15.6 %

^3^Standard error of mean

^4^N retention = N intake (g) - Fecal N (g) - Urinary N (g)

^5^N retention (%) = N retention/N intake × 100

^AB^ means with different superscripts in the same row significantly differ (*P* < 0.05)

^ab^means with different superscripts in the same row significantly differ (0.05 ≤ *P* < 0.10)

In nitrogen retention, N-intake was decreased gradually as dietary protein level decreased (CON: CP 18 %, P4(P5): CP 16.3 %, LP5: CP 15.6 %). Although N-feces did not show any significant difference, urinary nitrogen excretion was significantly decreased by 27 % with subdivision of phase feeding and reduction of dietary crude protein levels (*P* < 0.01). Furthermore, reduction of dietary crude protein level tended to show improvement (*P* = 0.08) in percentage of nitrogen retention by 33 %. Han et al. [15] represented that excretion of nitrogen through urine decreased by reducing dietary protein level. Similarly, Sutton et al. [37] indicated that reduction of dietary protein level in diet by 3 to 4 % resulted in decreasing the excretion of total nitrogen by approximately 28 to 36 %. Consequently, subdivision of phase feeding during growing period showed similar N-retention with non-divided groups even though subdivision group had lower N-intake. This result indicated that lower crude protein has benefits on the efficiency of nitrogen utilization in growing pigs.

### Carcass characteristics

The effect of dietary energy levels and phase feeding by protein levels on carcass characteristics was presented in Table 7. Generally, pork quality is directly related to crude fat contents in carcass and leaner pork resulted in lower water holding capacity, higher shear force as well as cooking loss [12]. However, there was no significant difference in moisture, crude protein, crude fat and crude ash of LM in current study. This result was in agreement with the observation of Jeong et al. [18] which demonstrated that phase feeding had no influence on the chemical compositions of longissimus muscle. In current study, difference of dietary energy level (100 kcal) and protein levels at each phase (0.8, 2.4, 1.1, 0.7, 0.4 %) among treatments did not affect the chemical composition of longissimus muscle in carcass. Water holding capacity was decreased by the effect of phase feeding through dietary protein levels (*P* < 0.01). There was an interaction between dietary energy levels and subdivision of phase through dietary protein levels (Table 7, *P* < 0.01). Cooking loss can be an indirect index of WHC because cooking loss decreased when water holding capacity had increased [16]. Similarly, cooking loss showed a tendency of increase by low dietary protein levels (*P* = 0.07). These results were in accordance with the research of Goerl et al. [12], who reported that increase of carcass fat content improved water holding capacity and Hamm [16], who demonstrated that relationship between water holding capacity and cooking loss. Several studies demonstrated that decreasing dietary protein level influenced to intramuscular fat [9, 12] and marbling of LM [2, 21]. Although crude fat content did not showed any difference by treatment effects, different protein level by phase feeding influenced to water holding capacity and cooking loss of LM. Beilken et al. [4] demonstrated that shear force was increased when water holding capacity decreased. However, no significant difference was observed in shear force of LM in current study. Also, Cromwell et al. [9] represented that shear force of loin were not significantly different from pigs fed low protein level and high protein level. Consequently, although chemical composition of LM was not significant differ among treatments, dietary protein levels had influence on WHC and cooking loss not shear force. When pigs were fed higher energy density feed, they tended to show thicker backfat thickness (P_2_ position) than pigs fed lower dietary energy density (P = 0.06). Increase of carcass backfat by high energy diet had been reported by several researchers [2, 9, 35]. In addition, tendency of reduction in backfat thickness was observed by subdivision of phase feeding or lowered dietary protein levels (*P* = 0.09). Similarly, Figueroa et al. [11] demonstrated that reducing dietary protein from 16 to 12 % increased backfat and Kerr et al. (1995) [21] indicated that pigs were fed low CP diet by 4 % compared to control, backfat thickness and 10th rib fat thickness were increased. However, Cromwell et al. [9] reported that backfat was not affected by dietary protein level and Lee et al. [26] represented that increasing the number of phase feeding regimes decreased the backfat thickness. Unfortunately, the effect of protein levels by phase feeding did not shown in current study, high energy diet increased backfat thickness and lower protein diet decreased backfat thickness.

**Table 7:** Effects of dietary energy and protein levels on carcass characteristics^1^

Criteria	Treatments						SEM^2^	*P*-value		
	ME 3,265	ME 3,365	CON	P4	P5	LP5		E	P	E×P
Proximate analysis, %										
Moisture	72.88	72.35	72.55	72.97	72.25	72.71	0.293	0.41	0.87	0.39
Crude protein	23.43	24.06	23.01	24.12	24.17	23.70	0.271	0.26	0.43	0.43
Crude fat	2.12	2.61	2.45	1.91	2.52	2.60	0.197	0.24	0.62	0.42
Crude ash	1.09	1.14	1.14	1.00	1.16	1.17	0.043	0.55	0.55	0.68
Physiochemical property										
Cooking loss^3^	30.63	30.49	29.81^b^	30.96^ab^	29.73^b^	31.75^a^	0.093	0.80	0.07	0.15
Shear force^4^	4.51	4.53	4.49	4.87	4.30	4.42	0.331	0.93	0.15	0.18
WHC^5^	55.49	56.01	56.43^A^	54.65^B^	56.87^A^	55.06^B^	0.310	0.17	0.01	0.01
Backfat thickness										
P_2_, mm	11.50^b^	11.96^a^	11.86^ab^	12.14^a^	11.64^ab^	11.29^b^	0.126	0.06	0.09	0.33

^1^Least squares means for three pigs per treatment

^2^Standard error of mean

^3^Cooking loss unit: %

^4^Shear force unit: kg/0.5 in

^5^WHC: water holding capacity

^AB^means with different superscripts in the same row significantly differ (*P* < 0.05)

^ab^means with different superscripts in the same row significantly differ (0.05 ≤ *P* < 0.10)

### Economical analysis

The effects of dietary energy levels and phase feeding by protein levels on feed cost, days to reaching 120 kg body weight, carcass yield and carcass price were presented in Table 8. There was significant difference in feed cost per weight gain during 0-3 week (*P* < 0.01), 6-9 week (*P* < 0.05) and overall period (*P* < 0.05) according to effect of dietary energy levels. Also, total feed cost per pig was affected by dietary energy levels during 6-9 week, 9-12 week, 12-13 week and overall period (*P* < 0.01). Increasing feed cost of high energy diets (3,365 kcal of ME/kg) is mainly caused by the soy oil supplementation.

**Table 8:** Effects of dietary energy and protein levels on economical efficiency

Criteria	Treatments						SEM^2^	*P*-value		
	ME 3,265	ME 3,365	CON	P4	P5	LP5		E	P	E×P
Feed cost per weight gain, won/kg										
0-3 weeks	778^B^	870^A^	851	847	831	768	17.2	0.01	0.33	0.63
3-6 weeks	988	1,049	1,029	1,010	1,048	985	19.1	0.15	0.75	0.66
6-9 weeks	1,035^B^	1,202^A^	1,124	1,120	1,180	1,050	41.1	0.02	0.60	0.34
9-12 weeks	1,119	1,186	1,166	1,188	1,172	1,083	32.4	0.51	0.62	0.63
12-13 weeks	1,110	1,177	1,318	1,136	998	1,121	54.2	0.98	0.14	0.38
0-13 weeks	5,029^B^	5,484^A^	5,489	5,302	5,230	5,006	88.8	0.02	0.17	0.33
Total feed cost per pig, won/head										
0-3 weeks	13,640	14,912	14,438	13,830	15,107	13,729	383.7	0.11	0.58	0.68
3-6 weeks	17,642	18,659	18,992	17,439	18,833	17,339	431.6	0.27	0.43	0.94
6-9 weeks	20,343^B^	22,140^A^	22,286	20,744	21,280	20,655	432.2	0.04	0.54	0.98
9-12 weeks	22,143^B^	24,288^A^	23,438	23,454	23,875	22,096	386.5	0.01	0.27	0.27
12-13 weeks	7,663^B^	9,151^A^	8,431	8,762	8,232	8,204	186.6	0.01	0.47	0.80
0-13 weeks	81,431^B^	89,150^A^	87,585	84,230	87,326	82,023	1469.7	0.01	0.55	0.92
Days to market weight (Day, reached 120 kg BW)										
	105.5	102.4	106.8	103.9	102.2	102.9	1.60	0.36	0.77	0.40
Carcass yield and price										
Carcass yield, %	76.7	76.7	77.2	76.0	76.2	77.4	0.270	0.95	0.23	0.99
Carcass price, 1,000 won	377.6^a^	368.4^b^	368.3	376.2	365.6	381.8	2.641	0.08	0.12	0.90

^1^Standard error of mean

^AB^means with different superscripts in the same row significantly differ (*P* < 0.05)

^ab^means with different superscripts in the same row significantly differ (0.05 ≤ *P* < 0.10)

Though there was no significant difference in feed cost per weight gain and total feed cost for market weight but numerical decrease of feed cost was observed when subdivision of phase feeding was increased and dietary crude protein level decreased. This result was partially agreed with the reports of Han et al. [14], Ko et al. (2004) [24] and Jeong et al. [18] which represented increasing subdivision of phases reduced the feed cost. There was no significant difference in days to market weight because growth performance was not affected by dietary treatments.

Carcass yield was not affected by the dietary energy levels and phase feeding by protein levels. However, when pigs were fed lower energy diet (3,265 kcal of ME/kg) tended to show higher carcass price than pigs fed higher energy diet (3,365 kcal of ME/kg) (*P* = 0.08).

### Conclusion

Dietary energy levels and subdivision of phase through dietary protein levels had no influence on growth performance. In growing 2 phase, Subdivision of growing period by lowered dietary protein levels showed the trend of nitrogen retention increase and reduced the BUN at 6 week by increasing nitrogen availability. However, dietary energy levels and subdivision of phase through dietary protein levels had no detrimental effect on carcass traits and pork quality. Consequently, phase feeding with low energy (3,265 kcal of ME/kg) and low protein diet (below the NRC req.) had no negative effect on growth performance, carcass characteristics but economical profits was improved due to high carcass price and low feed cost.

## Abbreviations

ADFI: Average daily feed intake
ADG: Average daily gain
BUN: Blood urea nitrogen
BW: Body weight
CP: Crude protein
CRD: Completely randomized design
LM: Longissimus muscle
ME: Metabolizable energy
RCBD: Randomized complete block design
WHC: Water holding capacity
